# Genomic Insights Into New Species of the Genus *Halomicroarcula* Reveals Potential for New Osmoadaptative Strategies in Halophilic Archaea

**DOI:** 10.3389/fmicb.2021.751746

**Published:** 2021-11-04

**Authors:** Ana Durán-Viseras, Cristina Sánchez-Porro, Antonio Ventosa

**Affiliations:** Department of Microbiology and Parasitology, Faculty of Pharmacy, University of Sevilla, Sevilla, Spain

**Keywords:** *Halomicroarcula*, haloarchaea, comparative genomic analysis, compatible solutes, *Halomicroarcula rubra* sp. nov., *Halomicroarcula nitratireducens* sp. nov., *Halomicroarcula salinisoli* sp. nov.

## Abstract

Metagenomic studies on prokaryotic diversity of hypersaline soils from the Odiel saltmarshes, South-west Spain, revealed a high proportion of genomic sequences not related to previously cultivated taxa, that might be related to haloarchaea with a high environmental and nutritional flexibility. In this study, we used a culturomics approach in order to isolate new haloarchaeal microorganisms from these hypersaline soils. Four haloarchaeal strains, designated strains F24A^T^, F28, F27^T^, and F13^T^, phylogenetically related to the genus *Halomicroarcula*, were isolated and characterized in detail. The phylogenomic tree based on the 100 orthologous single-copy genes present in the genomes of these four strains as well as those of the type strains of the species *Halomicroarcula pellucida* CECT 7537^T^, *Halomicroarcula salina* JCM 18369^T^ and *Halomicroarcula limicola* JCM 18640^T^, that were determined in this study, revealed that these four new isolates clustered on three groups, with strains F24A^T^ and F28 within a single cluster, and altogether with the species of *Halomicroarcula*. Additionally, Orthologous Average Nucleotide Identity (OrthoANI), digital DNA-DNA hybridization (dDDH) and Average Amino-acid Identity (AAI) values, likewise phenotypic characteristics, including their polar lipids profiles, permitted to determine that they represent three new species, for which we propose the names *Halomicroarcula rubra* sp. nov. (type strain F13^T^), *Halomicroarcula nitratireducens* sp. nov. (type strain F27^T^) and *Halomicroarcula salinisoli* sp. nov. (type strain F24A^T^). An in deep comparative genomic analysis of species of the genus *Halomicroarcula*, including their metabolism, their capability to biosynthesize secondary metabolites and their osmoregulatory adaptation mechanisms was carried out. Although they use a *salt-in* strategy, the identification of the complete pathways for the biosynthesis of the compatible solutes trehalose and glycine betaine, not identified before in any other haloarchaea, might suggest alternative osmoadaptation strategies for this group. This alternative osmoregulatory mechanism would allow this group of haloarchaea to be versatile and eco-physiologically successful in hypersaline environments and would justify the capability of the species of this genus to grow not only on environments with high salt concentrations [up to 30% (w/v) salts], but also under intermediate to low salinities.

## Introduction

Hypersaline environments are extreme habitats that have permitted the isolation and characterization of many halophilic microorganisms that have been used as models for the study of basic and applied purposes (Ventosa, 2006; Ventosa et al., 2015). These habitats are characterized by high levels of salinity, frequently accompanied by other extreme conditions, such as high or low temperature or pH values, UV radiation, hydrostatic pressure or high concentrations of toxic compounds (Rodríguez-Valera, 1988; Ventosa, 2006). To withstand these high salt concentrations and the constant fluctuations in salinity levels, halophilic microorganisms have developed diverse physiological adaptations (Galinski and Trüper, 1994; Kempf and Bremer, 1998; Gunde-Cimerman et al., 2018). The best adapted organisms to these high salt concentrations are prokaryotes belonging to the extremely halophilic archaea, members of the class *Halobacteria* (also called haloarchaea) (Amoozegar et al., 2017; Oren and Ventosa, 2017a). Although haloarchaea have been traditionally considered as a coherent group limited to use a *salt-in* osmoadaptation strategy, i.e., the accumulation of K^+^, Na^+^, and Cl^–^ ions, recent studies have brought to light their potential to employ additional alternative mechanisms (Anderson et al., 2011; Youssef et al., 2014).

Hypersaline environments are represented by a wide range of habitats, from aquatic or terrestrial to deep-sea, salt mines, salt-cured food or plants (Ventosa, 2006). However, hypersaline aquatic systems and more recently saline soils constitute the most extensively studied hypersaline environments (Ventosa et al., 2015). Traditionally, aquatic environments have been thoroughly studied, specially saline lakes and salterns (Ventosa et al., 2014, 2015; Oren, 2015), but the number of studies in hypersaline soils is much more reduced (Ventosa et al., 2008; Oren, 2011). Recent metagenomic studies carried out on hypersaline soils from the Odiel saltmarshes (South-west Spain) revealed a high proportion of genomic sequences not related to any cultivated organisms as well as a high environmental and nutritional flexibility of microorganism inhabiting these systems (Vera-Gargallo and Ventosa, 2018; Vera-Gargallo et al., 2019). With the purpose of isolating such of these metabolically diverse groups not isolated to date, we focused on these habitats by using culture-dependent methods. For that purpose, we used the current “culturomics” approach (Durán-Viseras et al., 2019a, 2021), in order to isolate new haloarchaeal groups taking advantage of the previous metagenomic studies, that showed an unexpected large proportion of haloarchaea in these hypersaline soils (Vera-Gargallo and Ventosa, 2018). As a result, four haloarchaeal strains phylogenetically related to the genus *Halomicroarcula* were isolated in pure culture.

The genus *Halomicroarcula* belongs to the family *Haloarculaceae*, within the order *Halobacteriales*, class *Halobacteria* and phylum *Euryarchaeota* (Echigo, 2016; Oren and Ventosa, 2017b). At the time of writing the genus *Halomicroarcula* comprises four species: *Halomicroarcula pellucida* (Echigo et al., 2013), which is the type species of the genus and was isolated from French marine salt, designated “Sel marin de Guérande,” *Halomicroarcula limicola* (Zhang and Cui, 2014) and *Halomicroarcula salina* (Zhang and Cui, 2015), both isolated from Yinggehai solar saltern, located in Hainan Province, China; and more recently, *Halomicroarcula amylolytica* (Chen et al., 2020), isolated from a salt mine in Yunnan Province, China. The genus *Halomicroarcula* includes Gram-stain-negative, motile and pleomorphic cells. They form non-pigmented and transparent or red-pigmented colonies. They are halophilic, neutrophilic and mesophilic. Other characteristics of this genus are their aerobic and heterotrophic metabolism (Echigo et al., 2013). Their major polar lipids are phosphatidylglycerol (PG), phosphatidylglycerolphosphate methyl ester (PGP-Me) and phosphatidylglycerol sulfate (PGS). Glycolipids, including sulfated mannosyl glucosyl diether (S-DGD-1) and mannosyl glucosyl diether (DGD-1) may be present in some species (Zhang and Cui, 2014; Echigo, 2016). The G + C content of strains of the genus *Halomicroarcula* range between 64.0 and 64.5 mol% (Echigo et al., 2013; Zhang and Cui, 2014; Echigo, 2016).

In this work, we have carried out an exhaustive taxogenomic study of the genus *Halomicroarcula*, and we have addressed the description of three new species of the genus *Halomicroarcula*. Besides, we have performed a comparative genomic analysis of species of this genus aimed at studying in depth their metabolism, their capacity to biosynthesize secondary metabolites and their osmoregulatory adaptation mechanisms that allow this group of microorganisms to be versatile and eco-physiologically successful in hypersaline environments.

## Materials and Methods

### Strains Isolation, Reference Strains and Culture Conditions

Strains F24A^T^, F28, F27^T^, and F13^T^ were isolated from saline soil samples (conductivity 54.5 CE_1__:__5_ mS/cm and pH 8.9) collected from the Odiel saltmarshes (Huelva, South-west Spain) (37°12′26″N 6°57′58″O). Samples were diluted, plated under sterile conditions and incubated at 37°C up to two months. Strains F24A^T^, F28, F27^T^, and F13^T^ were isolated and routinely grown in R2A medium (Difco) (pH adjusted to 7.5) supplemented with 25% (w/v) seawater salt solution prepared by dilution of SW 30% (w/v) stock solution containing (g/L): NaCl, 195; MgCl_2_⋅6H_2_O, 32.5; MgSO_4_⋅7H_2_O, 50.8; CaCl_2_, 0.83; KCl, 5.0; NaHCO_3_, 0.17; NaBr, 0.58. Purified agar (2%; Oxoid) was used as solidifying agent, when needed. For long term maintenance, cultures were preserved at –80°C in this medium containing 20% (v/v) glycerol (Durán-Viseras et al., 2021).

For taxonomic comparative purposes strains *Halomicroarcula pellucida* CECT 7537^T^, *Halomicroarcula salina* JCM 18369^T^ and *Halomicroarcula limicola* JCM 18640^T^, obtained from culture collections, were used in this study. These strains were also grown in the same medium and culture conditions as described above.

### DNA Extraction, Purification and Sequencing

The genomic DNA of strains F24A^T^, F28, F27^T^, F13^T^, *Halomicroarcula pellucida* CECT 7537^T^, *Halomicroarcula salina* JCM 18369^T^ and *Halomicroarcula limicola* JCM 18640^T^ was extracted and purified using the method described by Marmur (1961). DNA quality and concentration was checked by spectrophotometry (DeNovix DS-11 FX, DeNovix Technologies, Wilmington, Delaware, United States) and fluorometry (Qubit 3.0 Fluorometer, Thermofisher Scientific, United States). The 16S rRNA gene was amplified by PCR using the universal primers ArchF and ArchR (DeLong, 1992; Arahal et al., 1996), then cloned and sequenced as described previously (Durán-Viseras et al., 2019b). The *rpoB′* gene was amplified by PCR using the primers rpoBF and rpoBR (Fullmer et al., 2014). The genome of strains F13^T^, F24A^T^, F27^T^, F28, *Halomicroarcula pellucida* CECT 7537^T^, *Halomicroarcula limicola* JCM 18640^T^ and *Halomicroarcula salina* JCM 18369^T^ were sequenced using the Illumina HiSeq 4000 platform at StabVida (Oeiras, Portugal) and Novogene (Cambridge United Kingdom).

### Phylogenetic and Phylogenomic Analyses

Identification of phylogenetic neighbors and calculation of pairwaise 16S rRNA and *rpoB′* gene sequences similarities were conducted by using the Ez-BioCloud server (Yoon et al., 2017) and BLAST (Altschul et al., 1990), respectively. Additional 16S rRNA and *rpoB′* gene sequences, and genomes used for comparisons were retrieved from the GenBank/EMBL/DDBJ databases. Clustering of 16S rRNA and *rpoB′* gene sequences were determined using the neighbor-joining (Saitou and Nei, 1987) and maximum-likelihood (Felsenstein, 1981) algorithms implemented in the MEGA-X software (Kumar et al., 2018), using the Jukes-Cantor method (Jukes and Cantor, 1969) for evolutionary distances calculation. For phylogenetic tree branch support estimation, a bootstrap analysis based on 1000 replications was calculated (Felsenstein, 1985).

For phylogenomic analyses, core orthologous genes were identified from all analyzed genomes by an all-versus-all BLAST as implemented in the Enveomics collection toolbox (Rodriguez-R and Konstantinidis, 2016). As a result, a set of 100 conserved genes were retrieved and aligned using MUSCLE (Edgar, 2004). Phylogenomic tree of concatenated sequences was reconstructed by using the software MEGA-X (Kumar et al., 2018) with the neighbor-joining and maximum-likelihood methodology and Jukes-Cantor correction.

### Genome Assembly, Annotation and Comparative Genomics

The *de novo* assembly of the reads was performed using Spades 3.9.1 (Bankevich et al., 2012). Draft genome assembly was quality checked using Quast v2.3 (Gurevich et al., 2013). Genome completeness, contamination and strain heterogeneity was estimated using CheckM v1.0.5 (Parks et al., 2015).

Genome sequences were annotated with Prokka (Seemann, 2014). BlastKOALA (Kanehisa et al., 2016) was used to assign KO identifiers (K numbers) to orthologous genes present in the genomes and mapped to the KEGG pathways and KEGG modules to perform the metabolic pathways reconstructions.

CRISPR/Cas systems, prophage sequences and secondary metabolites were identified by the tools CRISPRCasFinder (Couvin et al., 2018), PHASTER (Zhou et al., 2011; Arndt et al., 2016) and antiSMASH 5.0 (Blin et al., 2019), respectively. Isoelectric points of predicted proteins were computed using the iep program from EMBOSS package (Rice et al., 2000). The Orthologous Average Nucleotide Identity (ANI), Average Amino-acid Identity (AAI), and digital DNA-DNA hybridization (dDDH) were calculated using the tools OAT v0.93.1 (Lee et al., 2016), AAI-Matrix (Rodriguez-R and Konstantinidis, 2016) and Genome-to-Genome Distance Calculator (GGDC) (Meier-Kolthoff et al., 2013), respectively.

### Phenotypic and Chemotaxonomic Characterization

Phenotypic features of strains F24A^T^, F28, F27^T^, and F13^T^ were performed according to the minimal standards established for the taxonomic description of novel taxa of the class *Halobacteria* (Oren et al., 1997) and following the methodology previously described by Durán-Viseras et al., 2020. *Halomicroarcula pellucida* CECT 7537^T^, *Halomicroarcula salina* JCM 18369^T^ and *Halomicroarcula limicola* JCM 18640^T^ were used as reference strains for taxonomic comparisons.

Comparative polar lipids analysis of strains F24A^T^, F28, F27^T^, and F13^T^ were determined by High-Performance Thin Layer Chromatography (HPTLC) as described elsewhere (Corral et al., 2015, 2016), using as spray reagents 5% H_2_SO_4_ (in water) or molybdenum blue. In this case, strains *Halomicroarcula pellucida* CECT 7537^T^, *Halomicroarcula salina* JCM 18369^T^, *Halomicroarcula limicola* JCM 18640^T^, *Halobacterium salinarum* DSM 3754^T^ and *Halorubrum saccharovorum* DSM 1137^T^ were used as reference species for polar lipids characterization. The polar lipids of strains F24A^T^, F28, F27^T^, F13^T^, *Halomicroarcula pellucida* CECT 7537^T^, *Halomicroarcula salina* JCM 18369^T^ and *Halomicroarcula limicola* JCM 18640^T^ were obtained from biomass cultured in R2A medium supplemented with 25% (w/v) seawater salt solution and pH adjusted to 7.5.

## Results and Discussion

### Phylogenetic and Phenotypic Analyses

Previous metagenomic studies on prokaryotic diversity of hypersaline soils showed a large proportion of haloarchaea, with a high percentage not closely related to any previously described taxa. For that reason, we isolated a large collection of strains from the Odiel saltmarsh hypersaline soils located in Huelva, South-west Spain, using different complex oligotrophic media and culture conditions, based on culturomics techniques as previously detailed (Durán-Viseras et al., 2021). Partial sequencing of the 16S rRNA gene permitted us to preliminary delineate the taxonomic position of the isolates. For this study we selected four new isolates, designated as strains F24A^T^, F28, F27^T^, and F13^T^, that were identified as members of the genus *Halomicroarcula*.

According to current practice, we determined the almost complete 16S rRNA gene sequences of strains F24A^T^, F28, F27^T^, and F13^T^. Similarly to other species of the genus *Halomicroarcula*, the four new strains showed two different copies of the 16S rRNA gene (designated as *rrnA* and *rrnB*), with sizes of 1441 bp and 1441 bp (for strain F13^T^), 1441 bp and 1442 bp (for strain F27^T^), 1446 bp and 1446 bp (for strain F24A^T^), and 1447 bp and 1446 bp (for strain F28), respectively. Their percentages of similarity with the type strains of species of *Halomicroarcula* (detailed in Supplementary Table 1), were in all cases equal or lower than 99.2% with *Halomicroarcula limicola* YGHS32^T^, 96.5% with *Halomicroarcula pellucida* BNERC31^T^ and 96.0% with *Halomicroarcula salina* YGHS18^T^. The phylogenetic tree generated on the basis of the 16S rRNA gene showed that they clustered within the *Halomicroarcula* branch (Supplementary Figure 1A). However, since this gene has been proved to be not very useful as a phylogenetic marker for haloarchaea, we also sequenced the *rpoB*′ gene, which has been recommended as an alternative for single-gene phylogenetic analyses (Enache et al., 2007; Minegishi et al., 2010). The phylogenetic tree obtained by the neighbor-joining method (Supplementary Figure 1B) showed a similar topology with the 16S rRNA gene tree, in which the four new strains were placed on the *Halomicroarcula* cluster, with strains F24A^T^ and F28 clustering together.

**FIGURE 1:**
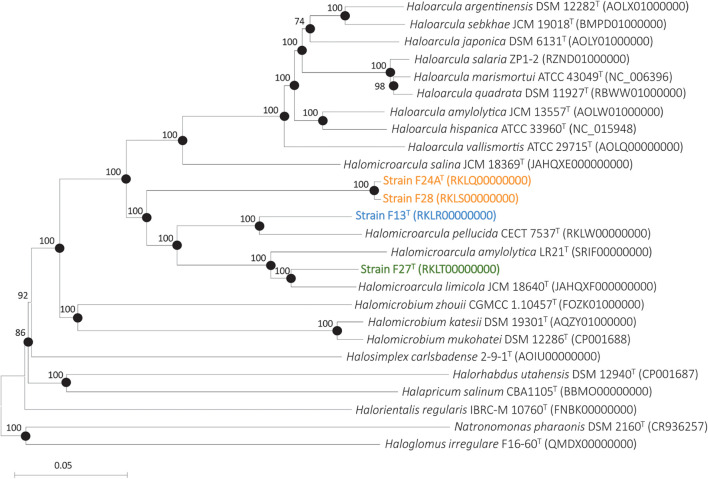
Neighbor-joining phylogenomic tree based on the concatenation of 100 orthologous single-copy genes shared by strains F13^T^, F24A^T^, F27^T^, F28, members of the genus *Halomicroarcula* and other related genera. Filled circles indicate branches that were also supported by the maximum-likelihood algorithm. Sequence accession number are shown in parentheses. Bootstrap values ≥70% are shown at branch points. Bar, 0.05 changes per nucleotide position.

In order to determine in more detail the phylogenomic relationships of these four new isolates with respect to the species of *Halomicroarcula* and according to the Minimal Standards recommendations (Chun et al., 2018), we sequenced their genomes as well as those of the type strains of the species *Halomicroarcula pellucida* CECT 7537^T^, *Halomicroarcula salina* JCM 18369^T^ and *Halomicroarcula limicola* JCM 18640^T^. Additionally, the genome of *Halomicroarcula amylolytica* LR21^T^, previously sequenced, was also included in this study. The main characteristics of these genomes are detailed in Table 1. All these genomic features are in accordance with the Minimal Standards established for the use of genomic data in prokaryotic taxonomy (Chun et al., 2018). The phylogenomic tree based on 100 orthologous single-copy genes present in all the genomes under study is shown in Figure 1. Bootstrap values of 100% in all branches related to *Halomicroarcula* supported this tree, generated by the neighbor-joining algorithm. This tree shows that the four new strains cluster within the *Halomicroarcula* branch, with strains F27^T^ and F13^T^ most closely related to *Halomicroarcula limicola* JCM 18640^T^ and *Halomicroarcula pellucida* CECT 7537^T^, respectively, while strains F24A^T^ and F28 clustered very close each other, suggesting that they may be members of the same species. Besides, this tree shows that the type strain of the species *Halomicroarcula salina* JCM 18639^T^ clustered with the genus *Haloarcula*, suggesting its taxonomic position should be revised.

**TABLE 1:** Main features of the sequenced genomes of strains F13^T^, F24A^T^, F28, F27^T^, and the type strains of species of *Halomicroarcula* used in this study.

Genomic feature	Strain F13^T^	Strain F24A^T^	Strain F28	Strain F27^T^	*Halomicroarcula limicola* JCM 18640^T^	*Halomicroarcula pellucida* CECT 7537^T^	*Halomicroarcula salina* JCM 18369^T^	*Halomicroarcula amylolytica* LR21^T^
Size (Mb)	4.7	4.0	4.1	5.2	3.9	3.9	3.8	5.0
Contigs	35	9	12	63	5	8	10	34
N50 (bp)	308161	1343505	742232	450140	1379950	1010699	594909	1435272
Completeness (%)	99.5	99.5	99.5	99.5	99.1	99.5	100	99.5
CDS	4781	4116	4189	5281	3888	4025	3827	5040
rRNA	6	5	3	3	4	3	2	4
tRNA	51	48	46	54	54	49	48	46
G + C (mol%)	64.4	64.1	63.9	63.2	65.9	65.5	66.7	62.0
Accession number	RKLR00000000	RKLQ00000000	RKLS00000000	RKLT00000000	JAHQXF000000000	RKLW00000000	JAHQXE000000000	SRIF00000000

**FIGURE 2:**
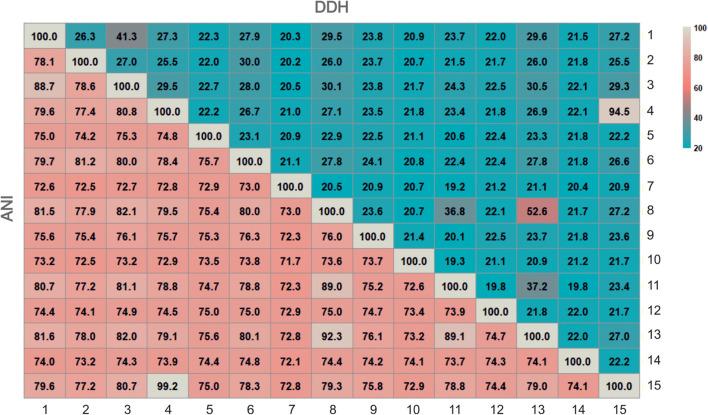
Heatmap of genome relatness among strains F13^T^, F24A^T^, F27^T^, F28, members of the genus *Halomicroarcula* and other related genera by means of OrthoANI and digital DDH. Strains: 1. *Halomicroarcula pellucida* CECT 7537^T^, 2. *Haloarcula vallismortis* ATCC 29715^T^, 3. Strain F13^T^, 4. Strain F24A^T^, 5. *Halosimplex carlsbadense* 2-9-1^T^, 6. *Halomicroarcula salina* JCM 18369^T^, 7. *Natronomonas pharaonis* DSM 2160^T^, 8. *Halomicroarcula limicola* JCM 18640^T^, 9. *Halomicrobium mukohatei* DSM 12286^T^, 10.*Halorhabdus utahensis* DSM 12940^T^, 11. *Halomicroarcula amylolytica* LR21^T^, 12. *Halorientalis regularis* IBRC-M 10760^T^, 13. Strain F27^T^, 14.*Halapricum salinum* CBA1105^T^, 15. Strain F28^T^. Genome accession numbers are indicated in Figure 1.

Currently, several Overal Genome Relatedness Indexes (OGRI) are used in order to measure similarities between genome sequences (Chun and Rainey, 2014). Many algorithms have been proposed for this purpose, but the three most widely used for taxonomic purposes are Orthologous Average Nucleotide Identity (OrthoANI) (Lee et al., 2016), and digital DNA-DNA hybridization (dDDH) (Meier-Kolthoff et al., 2013), especially useful for delineation at the species level, and Average Amino-acid Identity (AAI) (Rodriguez-R and Konstantinidis, 2016), for genus delineation. It is widely accepted that for delineation of prokaryotic species the threshold values for OrthoANI and dDDH are 95% and 70%, respectively (Konstantinidis and Tiedje, 2005; Auch et al., 2010; Chun and Rainey, 2014). However, for the delineation at the genus level there is no clear universal AAI cutoff value, with approximately 65% as a reference percentage (Konstantinidis et al., 2017). Figure 2 shows the OrthoANI and dDDH percentages calculated for all pairs of strains F24A^T^, F28, F27^T^, F13^T^, *Halomicroarcula pellucida* CECT 7537^T^, *Halomicroarcula salina* JCM 18369^T^, *Halomicroarcula limicola* JCM 18640^T^ and *Halomicroarcula amylolytica* LR21^T^, as well as for the type strains of other related haloarchaea. OrthoANI and dDDH values between strains F24A^T^ and F28 were 99.2% and 94.5%, respectively, confirming unequivocally that these two strains are members of the same species. On the other hand, their percentages with respect to the strains F27^T^ and F13^T^ and the type strains of species of *Halomicroarcula* and to other haloarchaea are equal or lower than 80.8% and 29.5%, respectively (Figure 2), which clearly indicates that they constitute a separated species of the genus *Halomicroarcula*. With respect to strains F27^T^ and F13^T^, their percentages of OrthoANI and dDDH with the most closely related species of *Halomicroarcula* are equal or lower than 92.3% and 52.6%, respectively (for strain F27^T^) and 88.7% and 41.3%, respectively (for strain F13^T^) (Figure 2), all of them values lower than those defined for the delineation of species, and thus supporting the new species status for both new isolates.

Concerning the AAI values between the four new strains and members of the genus *Halomicroarcula* and other related genera, they clearly confirm that these four new strains are members of the genus *Halomicroarcula*. Percentages of AAI of strains F24A^T^, F28, F27^T^, and F13^T^ among themselves and the species of *Halomicroarcula* are equal or higher than 71.3%, 71.5%, 72.7%, and 74.3%, respectively, values higher than the 65% generally accepted for the delineation at the genus level. It is noticeable that the AAI percentages for the new isolates and current species of the genus *Halomicroarcula* with *Haloarcula vallismortis* (type species of the genus *Haloarcula*) are 71.0%, 71.0%, 71.5%, and 73.1% for strains F24A^T^, F28, F27^T^, and F13^T^, respectively, and 73.9%, 79.6%, 72.0%, and 71.6%, respectively, for the species *Halomicroarcula pellucida* CECT 7537^T^, *Halomicroarcula salina* JCM 18369^T^, *Halomicroarcula limicola* JCM 18640^T^ and *Halomicroarcula amylolytica* LR21^T^. In fact, *Halomicroarcula salina* JCM 18369^T^ shows the higher percentage of AAI (79.6%) with *Haloarcula vallismortis* ATCC 29715^T^, and lower values with the remaining members of the genus *Halomicroarcula*: 71.3%, 71.5%, 73.5%, 74.3%, 74.5%, 74.2%, and 72.8% with strains F24A^T^, F28, F27^T^, and F13^T^ and the species *Halomicroarcula pellucida* CECT 7537^T^, *Halomicroarcula limicola* JCM 18640^T^ and *Halomicroarcula amylolytica* LR21^T^, respectively. Although the cutoff value of 65% for AAI delineation at the genus level is not universally accepted, considering these percentages of similarity, the species *Halomicroarcula salina* JCM 18369^T^ should be considered as a member of the genus *Haloarcula*. However, it must be noted that the AAI values for the remaining species of *Halomicroarcula* with respect to *Haloarcula vallismortis* ATCC 29715^T^ are also higher than 65% (71.6% for *Halomicroarcula amylolytica* LR21^T^, 72.0% for *Halomicroarcula limicola* JCM 18640^T^ and 73.9% for the type species of the genus, *Halomicroarcula pellucida* CECT 7537^T^). According to these results the species of the genera *Halomicroarcula* and *Haloarcula* should be merged into a single genus, as *Haloarcula*. A more exhaustive study, including a larger set of strains and based on additional genomic analysis reflecting their evolutionary relationships as well as their phenotypic features should be necessary in order to dilucidate the taxonomic status of the species of *Halomicroarcula* and *Haloarcula*.

We carried out a detailed phenotypic characterization of the four new isolates with respect to the type strains of species of *Halomicroarcula*, following the recommended minimal standards for describing new taxa of the class *Halobacteria* (Oren et al., 1997). These features included morphological, physiological, biochemical and nutritional characteristics, as well as the determination of the membrane polar lipids, which has been proved to be an important feature for the characterization of haloarchaeal genera (Oren et al., 1997, 2009). The results of the phenotypic features of the new isolates are shown in Supplementary Table 2 and in the new species descriptions included at the conclusion section. These data confirm that there are differential features supporting the proposal of the three new species of the genus *Halomicroarcula* (Supplementary Table 2). Concerning the polar lipids composition, they were analyzed by High-Performance Thin Layer Chromatography (HPTLC) and the results are shown in Supplementary Figure 2. The four new isolates, strains F24A^T^, F28, F27^T^, and F13^T^ showed the typical polar lipid profile of species of the genus *Halomicroarcula*, composed of phosphatidylglycerol (PG), phosphatidylglycerol phosphate methyl ester (PGP-Me), phosphatidylglycerol sulfate (PGS) and sulfated diglycosil diether (S-DGD-1) (Echigo et al., 2013; Zhang and Cui, 2014). Overall, all these phenotypic features are in agreement with the previous genomic results and support the placement of the new isolates into three new species of the genus *Halomicroarcula*.

### Genomic Features

In order to gain insights into the genome diversity of the genus *Halomicroarcula* we sequenced and analyzed the genome of the four strains isolated in this study and of the four already described species of the genus *Halomicroarcula*. The size of the genomes among the representatives of the genus *Halomicroarcula* ranged from 3.8 to 5.2 Mb, the DNA G + C content from 62.0 to 66.7 mol% and the total number of genes from 3827 to 5281. Most genomes of these strains ranged from 3.8 to 4.0 Mb, with the exception of three genomes that showed higher values (Table 1), even considering that the quality of these genomes are within the standards already described for prokaryotes (Chun et al., 2018).

On the other hand, several genetic elements detailed in Table 2 were also detected in those genomes. Members of the genus *Halomicroarcula* presented a large number of integrases and transposases, especially abundant in the isolated strains in comparison with the type strains of the previously described species of *Halomicroarcula*. Moreover, several CRISPR loci were also found, their number ranged from 2 in *Halomicroarcula salina* JCM 18369^T^ to 8 in strain F27^T^ (Table 2). While strains F24A^T^, F13^T^, F27^T^ and F28 presented *cas* cluster type IB and 94, 86, 84 and 74 spacers, respectively, not detectable *cas* genes were found in *Halomicroarcula limicola* JCM 18640^T^, *Halomicroarcula pellucida* CECT 7537^T^, *Halomicroarcula salina* JCM 18369^T^ and *Halomicroarcula amylolytica* LR21^T^ genomes, suggesting the lack of functionality of these systems in those strains. These data correlate with the high number of prophage sequences detected in *Halomicroarcula* genomes (Table 2), with a size between 5.4 and 33.1 kb. However, most of these prophage sequences were not complete.

**TABLE 2:** CRISPR loci and mobile genetic elements determined in the genomes of strains F13^T^, F24A^T^, F27^T^, F28 and the type strains of species of *Halomicroarcula*.

Strain	CRISPR loci	Prophage	Integrase	Transposase
Strain F13^T^	4	1	16	28
Strain F24A^T^	4	1	10	17
Strain F27^T^	8	0	9	29
Strain F28	6	2	12	12
*Halomicroarcula limicola* JCM 18640^T^	3	0	8	4
*Halomicroarcula pellucida* CECT 7537^T^	3	5	11	7
*Halomicroarcula salina* JCM 18369^T^	2	1	8	3
*Halomicroarcula amylolytica* LR21^T^	4	1	4	11

The presence of these elements in high copy numbers in the genomes of members of the genus *Halomicroarcula* reflect a high genomic plasticity of these strains (including genetic rearrangements or horizontal gene transfer events), particularly in those isolated in this study from hypersaline soils. This fact could suggest a great adaptation of these taxa to different ecological niches and their success in nature.

### Metabolism

Based on the genome annotation of members of the genus *Halomicroarcula* the major metabolic pathways of those strains could be reconstructed. For the carbohydrates uptake a large number of transporters were enconded in *Halomicroarcula* genomes reflecting their heterotrophic capabilities. Complete pathways involved in central carbohydrate metabolism (tricarboxylic acid cycle, oxidative pentose phosphate, Entner-Doudoroff or gluconeogenesis pathways) were present. However, in accordance with previous metabolic studies in haloarchaea (Falb et al., 2008; Anderson et al., 2011; Durán-Viseras et al., 2019a, 2021) the glycolysis pathway (Embden-Meyerhoff-Parnas) was truncated, therefore suggesting that alternative pathways like Entner-Doudoroff or the oxidative pentose phosphate could be used instead (Verhees et al., 2003). For the oxidation of the generated pyruvate to acetyl-CoA, both aerobic and anaerobic routes via pyruvate dehydrogenase and pyruvate ferredoxin oxidoreductase, respectively, were identified. Other carbohydrate metabolic pathways like the methylaspartate cycle, an anaplerotic acetate assimilation pathway, was also dilucidated in all *Halomicroarcula* genomes. This cycle has been previously identified in many other haloarchaea, such as the phylogenetically closest neighbor of the genus *Halomicroarcula*, the genus *Haloarcula* (Borjian et al., 2016). Its presence in haloarchaea has been frequently associated with the possession of genes for polyhydroxyalkanoate biosynthesis (Han et al., 2010; Borjian et al., 2016), which were also identified in *Halomicroarcula* genomes. Therefore, the presence of both pathways in *Halomicroarcula* genomes suggest the capability of members of this genus to biosynthetize polyhydroxialcanoates, as well as their adaptation for the assimilation of acetyl-CoA, produced from the internal carbon storage, during carbon starvation periods. This is a crucial factor for haloarchaea living under frequent starvation periods, such as on hypersaline soils from which our four strains were isolated. The genome of some members of the genus *Halomicroarcula* also encode for additional pathways related to carbohydrate metabolism (D-glucuronate and D-galacturonate degradation or glycogen biosynthesis), and with the hydrolysis of complex polysaccharides (α-amylase, chitinase, and endoglucanase enzymes), thus reflecting the metabolic diversity of representatives of this genus.

On the other hand, *Halomicroarcula* genomes encode the whole set of genes responsible for ammonia assimilation as a part of their nitrogen metabolism, such as enzymes for nitrate and nitrite reduction, the high-affinity ammonium transporter and glutamine synthetase and glutamate synthase. Moreover, complete biosynthesis pathways of several amino acids (i.e., arginine, cysteine, histidine, isoleucine, lysine, proline, serine, threonine, tryptophan, and valine) and few polyamine biosynthesis were identified along the genomes. Other determined sources of potential nitrogen rich compounds are different kinds of transporters for amino acids, urea and spermidine/putrescine uptake or a complete urease gene cluster.

Furthermore, *Halomicroarcula* genomes also encode genes for the ABC transporters for phosphate (pstSCAB) uptake, and in the case of *H. salina* JCM 18369^T^ and *H. pellucida* CECT 7537^T^ for phosphonate (phnCDE), as well as several mfs transporters related with multridrug efflux pump systems. Genes enconding archaella were also found in all *Halomicroarcula* genomes, confirming their motility as phenotypic feature.

In addition, rhodopsin-like sequences were also identified in members of the genus *Halomicroarcula*, suggesting the phototrophic capabilities of this group of prokaryotic microorganisms. Strains F13^T^, F24A^T^, F28 and *Halomicroarcula salina* JCM 18369^T^ presented sensory rhodopsins, haloarchaeal proton pumps and halorhodopsins, while *Halomicroarcula pellucida* CECT 7537^T^ exhibited sensory rhodopsin and haloarchaeal proton pump, and strain F27^T^ and *Halomicroarcula amylolytica* LR21^T^ only sensory rhodopsins. No rhodopsin-like sequences were identified in *Halomicroarcula limicola* JCM 18640^T^. The presence of rhodopsin like-sequences in most *Halomicroarcula* genomes suggest a versatile metabolic flexibility in illuminated conditions for members of this genus. These results are in accordance with recent comparative genomic studies in other haloarchaeal genera (Durán-Viseras et al., 2019a, 2020, 2021) in which rhodopsins were also identified, and also with previous metagenomic analyses on hypersaline systems (Fernández et al., 2014; Vera-Gargallo and Ventosa, 2018) that showed the existence of a large number of rhodopsin coding genes, clearly indicating the wide use of light by haloarchaea in these extreme habitats.

Regarding vitamins and cofactors metabolism the complete pathway for the vitamin B_12_ biosynthesis was determined in all members of the genus *Halomicroarcula*, suggesting the capability of this genus for its *de novo* synthesis. Remarkably, this pathway was recently identified by genomic studies in other haloarchaeal members of the genus *Halonotius* (Durán-Viseras et al., 2019a). An ABC transporter for biotin uptake was also encountered in *Halomicroarcula* genomes.

Besides, as a part of the metabolic analyses we also searched for metal resistance genes in *Halomicroarcula* genomes. While *Halomicroarcula salina* JCM 18369^T^ and *Halomicroarcula amylolytica* LR21^T^ encode the complete arsenite resistance gene cluster (*ars*), this cluster was truncated in the other *Halomicroarcula* members. However, strains isolated in this study (F13^T^, F24A^T^, F27^T^, and F28) and *Halomicroarcula amylolytica* LR21^T^ encode the CzcD transporter, a member of the cation diffusion facilitator (CDF) protein family, which was absent in the other *Halomicroarcula* reference strains. This transporter not only reflects a heavy metal resistance against cobalt, zinc or cadmium, but has also been suggested as a biomarker of nickel and vanadium pollution in some *Bacteria* (Anton et al., 1999; Fierros-Romero et al., 2020). On the contrary, none of the genes involved in copper or mercury resistance were identified in any of the studied genomes. These results could be related to the different stress conditions affecting the diverse ecological niches that they inhabit. In addition, several ABC metal transporters for zinc, cobalt and nickel were identified, which display a less critical role in maintaining metal homeostasis than CDF family transporters (Kaur et al., 2006).

### Osmoadaptative Capabilities

To cope with the high salt concentrations and salinity fluctuations of hypersaline environments, halophilic microorganisms have developed diverse mechanisms of adaptation (Gunde-Cimerman et al., 2018). In order to delucidate the osmorregulatory strategy employed by *Halomicroarcula* members and their closest phylogenetic neighbors (the genera *Haloarcula* and *Halomicrobium*), their proteome was analyzed and compared with those of *salt-in* microorganisms [i.e., *Haloquadratum walsbyi* C23^T^ (Dyall-Smith et al., 2011) and *Salinibacter ruber* DSM 13855^T^ (Mongodin et al., 2005)] and with that of a *salt-out* bacterium [*Spiribacter salinus* M19-40^T^ (López-Pérez et al., 2013)] (Supplementary Figure 3). In accordance to *Haloquadratum walsbyi* C23^T^ and *Salinibacter ruber* DSM 13855^T^ and by contrast with *Spiribacter salinus* M19-40^T^, *Halomicroarcula* representatives exhibited an acidic proteome with a low isoelectric point peak around 4.0 (Supplementary Figure 3A). Hence, highlighting a typical *salt-in* strategy for all members of this genus. Similar results were obtained for *Haloarcula* and *Halomicrobium* representatives (Supplementary Figure 3B). Besides, during the indeep genomic analysis of members of the genus *Halomicroarcula* we investigated the presence of genes putatively involved in osmorregulation. In conformity with results mentioned above, several transporters for Na^+^ extrusion, K^+^ uptake and Cl^–^ homeostasis were also identified, reinforcing its *salt-in* strategy.

According to previous suggestions (Youssef et al., 2014) and considering the frequent periods of low or fluctuenting salinity levels of the saline soils from which strains F13^T^, F24A^T^, F27^T^, and F28 were isolated, it is not rare that they could not exclusively employ a *salt-in* strategy. Noteworthy, genes encoding *de novo* synthesis of trehalose via trehalose-6-phosphate synthase (OtsA) and trehalose-6-phosphatase (OtsB) were encountered in the genomes of strains F24A^T^, F27^T^, F28, *Halomicroarcula amylolytica* LR21^T^ and *Halomicroarcula limicola* JCM 18640^T^ suggesting their capability for trehalose biosynthesis. Trehalose is a dissacharide which has been reported to be used as an osmolyte by different organisms (Shivanand and Mugeraya, 2011). On the other side, the *otsAB* pathway was not found complete for strains F13^T^, *Halomicroarcula salina* JCM 18369^T^ and *Halomicroarcula pellucida* CECT 7537^T^, which lack the OtsB enzyme. However, the genome of strain F13^T^ was the only one exhibiting the enzyme trehalose synthase (TreT) suggesting this is the pathway used by this strain to produce trehalose. Although the synthesis of the compatible solute trehalose has been previously reported for other members of the haloarchaea (Youssef et al., 2014), no evidence has been demonstrated for any *Haloarcula* representatives (Youssef et al., 2014), the phylogenetically closest neighbors of the genus *Halomicroarcula*. Additionally, the presence of genes encoding the different trehalose biosynthesis pathways were analyzed during the course of this study in all members of the genera *Haloarcula* and *Halomicrobium* with available genomes (Supplementary Table 3). The enzyme trehalose synthase (TreT) was not found in any *Haloarcula* or *Halomicrobium* genomes, whereas the OtsAB pathway was only found complete in *Halomicrobium zhouii* CGMCC 1.10457^T^ (only OtsA was present in *Haloarcula sebkhae* JCM 19018^T^, *H. salaria* ZP1-2, *H. quadrata* DSM 11927^T^, *H. marismortui* ATCC 43049^T^, *H. hispanica* ATCC 33960^T^ and *H. argentinensis* DSM 12282^T^ genomes; while *Halomicrobium katesii* DSM 19301^T^ only possessed OtsB). These results suggest that the ability to synthethize trehalose is not extended neither in the genera *Haloarcula* and *Halomicrobium*, in accordance with previous presumptions of Youssef et al. (2014) for the genus *Haloarcula*. The ability to synthethize trehalose has been previously described for members of the class *Halobacteria* dwelling environments with lower salinity or salinity fluctuations (Youssef et al., 2014), such as the hypersaline soil from which strains F13^T^, F24A^T^, F27^T^ and F28 were isolated.

Surprinsingly, the complete pathway for the biosynthesis of the compatible solute glycine betaine from choline, was identified in the genomes of strain F13^T^ and *Halomicroarcula limicola* JCM 18640^T^. No evidencies were identified in any of the genomes from species of the genera *Haloarcula* or *Halomicrobium*. Accordingly, the BCCT family transporters: OpuD and BetT, possibly for glycine betaine uptake or for other types of compatible solutes (Ziegler et al., 2010), were also present in the genomes of strains F13^T^, F27^T^, *Halomicroarcula pellucida* CECT 7537^T^, *Halomicroarcula amylolytica* LR21^T^ and *Halomicroarcula salina* JCM 18369^T^ (in the case of OpuD) and strains F13^T^, F27^T^, *Halomicroarcula limicola* JCM 18640^T^ and *Halomicroarcula pellucida* CECT 7537^T^ (in the case of BetT). In the same way, OpuD was also present in *Haloarcula sebkhae* JCM 19018^T^, *H. argentinensis* DSM 12282^T^ and *Halomicrobium zhouii* CGMCC 1.10457^T^ genomes, and BetT in the genomes of *Haloarcula sebkhae* JCM 19018^T^ and *H. argentinensis* DSM 12282^T^. Moreover, *Halomicroarcula salina* JCM 18369^T^ and *Halomicroarcula amylolytica* LR21^T^ also showed the ABC transporter OpuA for betaine incorporation (Kempf and Bremer, 1995). Small conductance mechanosensitive channels from the MscS family were identified as well in *Halomicroarcula* genomes, and in the genomes of few members of the genera *Haloarcula* and *Halomicrobium*. These systems are ubiquitously used by microorganisms to manage the rapid transition from high salinity surroundings to environments with moderate salinities (Booth and Blount, 2012). The functionality of such safety valves has been demonstrated in some *Archaea* such as the marine thaumarchaeon *Nitrosopumilus maritimus* (Widderich et al., 2016). While BCCT family transporters are quite common in members of the class *Halobacteria* (Anderson et al., 2011; Youssef et al., 2014; Gunde-Cimerman et al., 2018), to the best of the authors knowledge, this is the first time that genes encoding glycine betaine synthesis are reported for any haloarchaea by far. This fact, could indicate an additional osmoadaptative mechanism for strains of the genus *Halomicroarcula*, which would corroborate the versatility of this group of microorganisms to adapt to environments with different salinities. The large amount of genes related with glycine betaine in the genomes of the genus *Halomicroarcula* raises the possibility that this organic solute could play an important role as osmoprotectant in this archaeal group that needs to be further investigated.

### Biosynthesis of Secondary Metabolites

**FIGURE 3:**
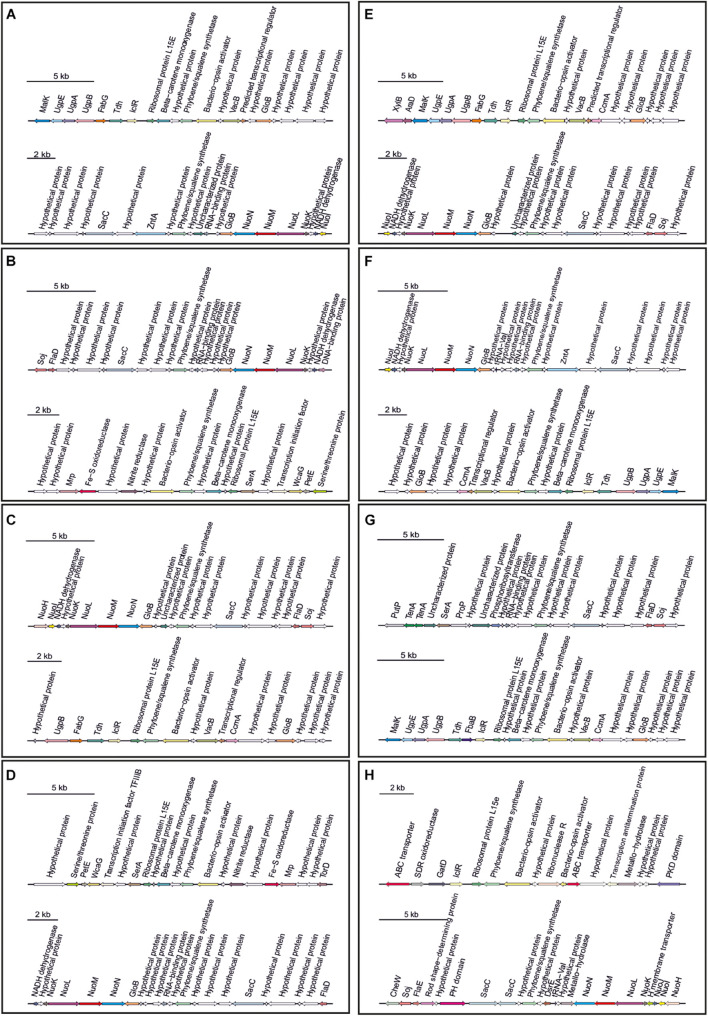
Terpene biosynthetic gene clusters identified in *Halomicroarcula* genomes: **(A)** Strain F13^T^, **(B)** Strain F24A^T^, **(C)** Strain F27^T^, **(D)** Strain F28, **(E)** *Halomicroarcula limicola* JCM 18640^T^, **(F)** *Halomicroarcula pellucida* CECT 7537^T^, **(G)** *Halomicroarcula salina* JCM 18369^T^, and **(H)** *Halomicroarcula amylolytica* LR21^T^.

**FIGURE 4:**
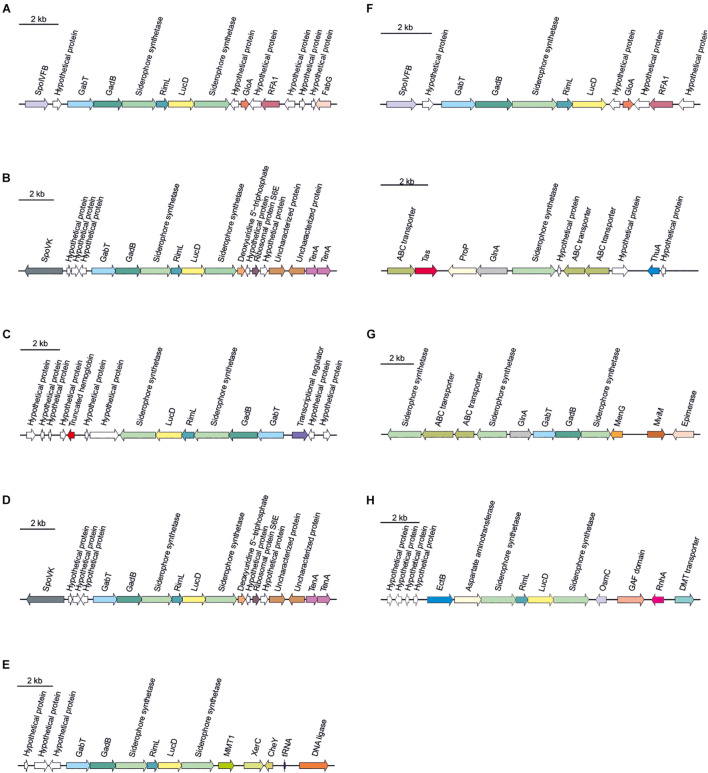
Siderophore biosynthetic gene clusters identified in *Halomicroarcula* genomes: **(A)** Strain F13^T^, **(B)** Strain F24A^T^, **(C)** Strain F27^T^, **(D)** Strain F28, **(E)** *Halomicroarcula limicola* JCM 18640^T^, **(F)** *Halomicroarcula pellucida* CECT 7537^T^, **(G)** *Halomicroarcula salina* JCM 18369^T^, and **(H)** *Halomicroarcula amylolytica* LR21^T^.

**FIGURE 5:**
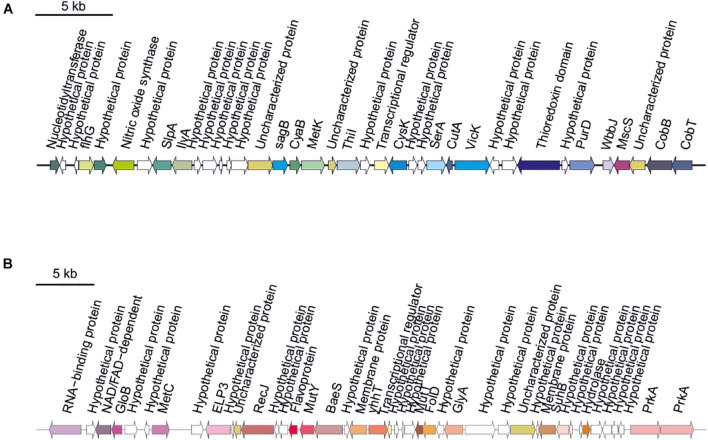
Thiopeptide biosynthetic gene clusters identified in *Halomicroarcula* genomes: **(A)** Strain F24A^T^ and **(B)** *Halomicroarcula pellucida* CECT 7537^T^.

**FIGURE 6:**
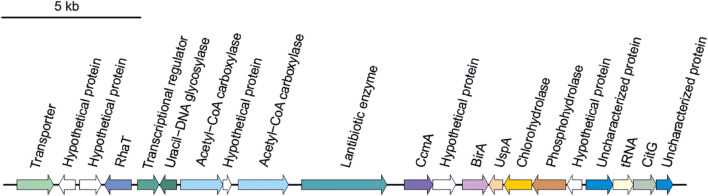
Lanthipeptide biosynthetic gene cluster identified in *Halomicroarcula salina* JCM 18369^T^.

Secondary metabolites are a range of bioactive compounds of high interest for biotechnological, pharmaceutical or industrial applications (Charlesworth and Burns, 2015). The production of secondary metabolites give some environmental advantages to the microorganisms producing them (such as tolerance against environmental stress or interspecies defenses) (Osbourn, 2010; Piasecka et al., 2015; Wang and Lu, 2017; Wang et al., 2019). While secondary metabolites in the domains *Bacteria* and *Eukarya* have been deeply studied, analysis of these compounds in *Archaea* are more scarce (Charlesworth and Burns, 2015; Corral et al., 2020). As a part of our genomic analysis we also investigated the presence of genes involved on the biosynthesis of secondary metabolites in the genomes of members of the genus *Halomicroarcula*. The clusters that we have identified on these genomes are detailed in Table 3 and Figures 3–6. Two different terpene clusters were detected in all *Halomicroarcula* genomes, which shared similarities within the different analyzed strains (Figure 3). To the best of the authors knowledge the presence of terpenes in *Archaea* has only been reported in a previous study (Wang et al., 2019), which also indicated the presence of two terpene clusters in the *Halobacteria* genomes. Terpenes are metabolites widely distributed in plants, fungi and bacteria in which they play a protective role; these compounds have also been suggested to be a source for the discovery of natural products (Pichersky et al., 2006; Yamada et al., 2015). On the other hand, siderophores, iron-chelating molecules produced during stress conditions or iron deficiency (Srivastava and Kowshik, 2013), were also distributed in the seven analyzed genomes of members of the genus *Halomicroarcula* (Figure 4). Despite the ubiquity of iron in the environment its solubility is very low, leading to the siderophore strategy to overcome this scarcity. The production of siderophores has also been detected before in some other haloarchaea (Dave et al., 2006). Finally, we detected a thiopeptide cluster in the genomes of strain F24A^T^ and *Halomicroarcula pellucida* CECT 7537^T^ (Figure 5), and a lanthipeptide in the genome of *Halomicroarcula salina* JCM 18369^T^ (Figure 6), both of them members of the ribosomally synthesized and post-translationally modified peptides (RiPP) family of natural products. No similarity was observed between the identified thiopeptide clusters (Figure 5). While thiopeptides constitute important components of the defense system in archaea, lanthipeptides display different biological activities, such as antimicrobial or antiallodynic (Repka et al., 2017; Wang and Lu, 2017). Thus, the biosynthesis of this wide variety of secondary metabolites by strains of the genus *Halomicroarcula* suggests the versatility of members of this genus, which have developed several ecological advantages such as a great adaptability to extreme conditions or defense mechanisms. In addition, our results brought to light their possible applications on the biotechnological field as a source of new natural compounds, also recently proposed for other novel haloarchaeal taxa (Durán-Viseras et al., 2021).

**TABLE 3:** Number of genetic clusters involved in the biosynthesis of secondary metabolites determined in the genomes of strains F13^T^, F24A^T^, F28, F27^T^, and the type strains of species of *Halomicroarcula*.

Cluster type	F13^T^	F24A^T^	F28	F27^T^	*Halomicroarcula limicola* JCM 18640^T^	*Halomicroarcula pellucida* CECT 7537^T^	*Halomicroarcula salina* JCM 18369^T^	*Halomicroarcula amylolytica* LR21^T^
Terpene	2	2	2	2	2	2	2	2
Siderophore	1	1	1	1	1	2	1	1
Lanthipeptide	0	0	0	0	0	0	1	0
Thiopeptide	0	1	0	0	0	1	0	0

## Conclusion

The indeep comparative genomic analysis of the genus *Halomicroarcula* brought to light the presence of integrases, transposases and other gene transfer systems in high copy in the studied genomes, suggesting a vast plasticity for genes adquisition in members of the genus *Halomicroarcula*. This genomic plasticity leads to the versatile metabolism observed in the studied strains, such as the presence of metal resistance genes and genes coding for diverse carbohydrates pathways, some of them (i.e., methylaspartate cycle and polyhydroxyalkanoate biosynthesis) advantageous during carbon starvation periods. Besides, the capacity to biosynthethize diverse secondary metabolites (i.e., terpenes, siderophores, lanthipeptides or thiopeptides), could provide an ecological benefit for these microorganisms such as defense systems or adjustability to limiting conditions, and could also be source of novel compounds for biotechnological applications. The analysis of the proteome of members of the genus *Halomicroarcula* indicate they use a *salt-in* strategy. Nevertheless, complete pathways for the biosynthesis of compatible solutes (i.e., trehalose and glycine betaine), identified for the first time in haloarchaea during the detailed genomic analysis carried out in this study, suggests that alternative osmoadaptation strategies could be additionally employed. All these facts could give an ecological advantage for these microorganisms, which provide the genus *Halomicroarcula* the basis for the adaptation to a wide range of ecological niches and hence, playing a crucial role in the ecophysiological success of this taxa in the nature. Besides, this fact might justify the ability of these haloarchaea to grow at intermediate to low salinity environments.

On the other side, the exhaustive taxogenomic and phenotypic study carried out in this work, has permitted the characterization and description of three new species within the genus *Halomicroarcula*, for which we propose the new names *Halomicroarcula rubra* sp. nov., *Halomicroarcula nitratireducens* sp. nov. and *Halomicroarcula salinisoli* sp. nov.; and whose descriptions are detailed below.

### *Halomicroarcula rubra* sp. nov.

*Halomicroarcula rubra* (ru′bra. L. fem. adj. *rubra*, red).

Cells are Gram-stain-negative, motile rods with 1 × 1.2-2.5 μm. Does not grow anaerobically with L-arginine, dimethyl sulfoxide (DMSO) or potassium nitrate. Colonies are circular, entire, red pigmented with 0.2–0.3 mm in diameter on R2A25 medium after 14 days of incubation at 37°C. Extremely halophilic, able to grow in media with 10–30% (w/v) salts, with optimal growth at 25–30% (w/v) salts. No growth occurs in the absence of NaCl. Mg^2+^ is not required for growth. Able to grow in the pH range of 6.0–9.0 and from 25 to 50°C, with optimal growth at pH 7.5–8.0 and at 37°C. Chemoorganotrophic and aerobic. Catalase positive and oxidase negative. Gelatin is hydrolyzed but starch, Tween 80 and aesculin are not. Nitrate and nitrite are reduced, without gas production. H_2_S is produced but indole and urease are not. Methyl red test is positive. Voges-Proskauer is negative. Acid is produced from D-arabinose, arbutin, L-citrulline, D-fructose, glycerol, D-glucose, D-ribose, L-xylitol and D-xylose but not from D-amygdalin, D-cellobiose, dulcitol, D-galactose, lactose, D-maltose, D-mannitol, D-mannose, D-melezitose, D-raffinose, D-sucrose, sorbitol or D-trehalose. The following compounds are used as carbon and energy source: D-melibiose, L-arginine, and L-methionine. The following compounds are not used as sole carbon and energy source: D-arabinose, D-cellobiose, fructose, D-galactose, D-glucose, lactose, maltose, D-mannose, L-raffinose, ribose, sucrose, D-trehalose, D-xylose, D-melezitose, salicin, butanol, dulcitol, ethanol, glycerol, D-mannitol, D-sorbitol, xylitol, methanol, benzoate, citrate, formate, fumarate, propionate, valerate, hippurate, malate, pyruvate, tartrate, L-alanine, L-cysteine, glutamine, L-glycine, L-lysine, isoleucine or valine. The major polar lipids are phosphatidylglycerol (PG), phosphatidylglycerol phosphate methyl ester (PGP-Me), phosphatidylglycerol sulfate (PGS) and sulfated mannosyl glucosyl diether (S-DGD-1). The DNA G + C content is 64.4 mol% (genome).

The type strain is F13^T^ (= CCM 8888^T^ = CECT 9686^T^ = IBRC-M 11249^T^ = JCM 33313^T^), isolated from a hypersaline soil located in Odiel saltmarshes, Huelva, Spain.

The GenBank/EMBL/DDBJ accession number for the 16S rRNA and *rpoB*′ gene sequences of *Halomicroarcula rubra* F13^T^ are MH447277 (*rrnA* gene), MH447279 (*rrnB* gene) and MH454085, respectively, and that of the complete genome is RKLR00000000.

### *Halomicroarcula nitratireducens* sp. nov.

*Halomicroarcula nitratireducens* (ni.tra.ti.re.du’cens. N.L. masc. n. *nitras* (gen. *nitratis*), nitrate; L. pres. part. *reducens*, converting to a different state, reducing; N.L. part. adj. *nitratireducens*, reducing nitrate).

Cells are Gram-stain-negative, motile rods with 1 × 1.2–2.5 μm. Does not grow anaerobically with L-arginine, DMSO or potassium nitrate. Colonies are circular, entire, red to orange pigmented with 0.2–0.3 mm in diameter on R2A25 medium after 14 days of incubation at 37°C. Extremely halophilic, able to grow in media with 10–30% (w/v) salts, with optimal growth at 25–30% (w/v) salts. No growth occurs in the absence of NaCl. Mg^2+^ is not required for growth. Able to grow in the pH range of 6.0–9.0 and from 25 to 50°C, with optimal growth at pH 7.5 and at 37°C. Chemoorganotrophic and aerobic. Catalase and oxidase negative. Starch and aesculin are hydrolyzed but gelatin and Tween 80 are not. Nitrate and nitrite are reduced, without gas production. H_2_S, indole and urease are not produced. Methyl red test is positive. Voges-Proskauer is negative. Acid is produced from D-arabinose, arbutin, L-citrulline, D-fructose, D-glucose, D-ribose and D-xylose but not from D-amygdalin, D-cellobiose, dulcitol, D-galactose, glycerol, lactose, D-maltose, D-mannitol, D-mannose, D-melezitose, D-raffinose, D-sucrose, sorbitol, D-trehalose or L-xylitol. The following compounds are used as carbon and energy source: fructose, D-galactose, D-glucose, maltose, ribose, sucrose, salicin, glycerol, D-mannitol, D-sorbitol, methanol, fumarate, or L-lysine. The following compounds are not used as sole carbon and energy source: D-arabinose, D-cellobiose, lactose, D-xylose, butanol, dulcitol, ethanol, xylitol, benzoate, citrate, formate, propionate, valerate, hippurate, malate, pyruvate, tartrate, L-alanine, L-arginine, L-cysteine, glutamine, L-methionine, L-glycine, isoleucine or valine. The major polar lipids are phosphatidylglycerol (PG), phosphatidylglycerol phosphate methyl ester (PGP-Me), phosphatidylglycerol sulfate (PGS) and sulfated mannosyl glucosyl diether (S-DGD-1). The DNA G + C content is 63.2 mol% (genome).

The type strain is F27^T^ (= CCM 8887^T^ = CECT 9636^T^ = IBRC-M 11233^T^ = JCM 33314^T^), isolated from a hypersaline soil located in Odiel saltmarshes, Huelva, Spain.

The GenBank/EMBL/DDBJ accession number for the 16S rRNA and *rpoB*′ gene sequences of *Halomicroarcula nitratireducens* F27^T^ are MH447286 (*rrnA* gene), MH447284 (*rrnB* gene) and MH454093, respectively, and that of the complete genome is RKLT00000000.

### *Halomicroarcula salinisoli* sp. nov.

*Halomicroarcula salinisoli* (sa.li.ni.so’li. N.L. masc. adj. *salinus*, salty; L. neut. n. *solum*, soil; N.L. gen. n. *salinisoli*, of salty soil).

Cells are Gram-stain-negative, motile, pleomorphic rods with 1 × 1.2–2.5 μm. Does not grow anaerobically with L-arginine, DMSO or potassium nitrate. Colonies are circular, entire, pink pigmented with 0.2–0.3 mm in diameter on R2A25 medium after 14 days of incubation at 37°C. Extremely halophilic, able to grow in media with 15–30% (w/v) salts, with optimal growth at 25% (w/v) salts. No growth occurs in the absence of NaCl. Mg^2+^ is not required for growth. Able to grow in the pH range of 6.0–8.5 and from 25 to 50°C, with optimal growth at pH 7–7.5 and at 37°C. Chemoorganotrophic and aerobic. Catalase positive and oxidase negative. Gelatin, aesculin and Tween 80 are hydrolyzed but starch is not. Nitrate and nitrite are reduced, without gas production. H_2_S production is variable, indole and urease are not produced. Methyl red test is positive. Voges-Proskauer is negative. Acid is produced from D-arabinose, arbutin, D-cellobiose, L-citrulline, D-fructose, D-glucose, D-ribose and D-xylose but not from dulcitol, D-galactose, lactose, D-maltose, D-mannitol, D-mannose, D-melezitose, D-raffinose, D-sucrose, sorbitol, D-trehalose or L-xylitol. The following compounds are used as carbon and energy source: D-cellobiose, D-glucose, maltose, sucrose, D-sorbitol, citrate, fumarate or tartrate. The following compounds are not used as sole carbon and energy source: D-arabinose, D-galactose, lactose, ribose, D-xylose, salicin, glycerol, xylitol, benzoate, propionate, valerate, hippurate, pyruvate, L-arginine, L-cysteine, L-methionine, isoleucine or valine. The major polar lipids are phosphatidylglycerol (PG), phosphatidylglycerol phosphate methyl ester (PGP-Me), phosphatidylglycerol sulfate (PGS) and sulfated mannosyl glucosyl diether (S-DGD-1). The DNA G + C content is 63.9–64.1 mol% (genome).

The type strain is F24A^T^ (= CCM 8955^T^ = CECT 9687^T^), isolated from a hypersaline soil located in Odiel saltmarshes, Huelva, Spain. The DNA G + C content of the type strain is 64.1 mol% (genome).

The GenBank/EMBL/DDBJ accession number for the 16S rRNA and *rpoB*′ gene sequences of *Halomicroarcula salinisoli* F24A^T^ are MH447282 (*rrnA* gene), MH447281 (*rrnB* gene) and MH454092, respectively, and that of the complete genome is RKLQ00000000.

An additional strain of this species is strain F28. The DNA G + C content of this strain is 63.9 mol% (genome). The GenBank/EMBL/DDBJ accession number for the 16S rRNA and *rpoB*′ gene sequences of this strain are MH450228 (*rrnA* gene), MH447330 (*rrnB* gene) and MH454094, respectively, and that of the complete genome is RKLS00000000.

## Data Availability Statement

The datasets presented in this study can be found in online repositories. The names of the repository/repositories and accession number(s) can be found below: https://www.ncbi.nlm.nih.gov/genbank/, the 16S rRNA and *rpoB*′ genes and the genome sequences generated for this study can be found in the GenBank/EMBL/DDBJ database under the accession numbers MH447277, MH447279, MH447282, MH447281, MH450228, MH447330, MH447286, MH447284, MH454085, MH454092, MH454094, MH454093, RKLR00000000, RKLQ00000000, RKLS00000000, RKLT00000000, JAHQXF000000000, RKLW00000000, and JAHQXE000000000.

## Author Contributions

AD-V, CS-P, and AV did the conceptualization. AD-V did the strains isolation, the taxogenomic characterization, and the comparative genomic analyses. AD-V and AV wrote the manuscript. AD-V prepared the tables and figures. AV and CS-P did the funding acquisition. All authors read and approved the final version of the manuscript.

## Conflict of Interest

The authors declare that the research was conducted in the absence of any commercial or financial relationships that could be construed as a potential conflict of interest.

## Publisher’s Note

All claims expressed in this article are solely those of the authors and do not necessarily represent those of their affiliated organizations, or those of the publisher, the editors and the reviewers. Any product that may be evaluated in this article, or claim that may be made by its manufacturer, is not guaranteed or endorsed by the publisher.
