# Supplementary material for: Genomic Insights Into New Species of the Genus Halomicroarcula Reveals Potential for New Osmoadaptative Strategies in Halophilic Archaea
Source: Front Microbiol. 2021 Nov 4;12:751746. doi: 10.3389/fmicb.2021.751746 (PMC8600319; doi:10.3389/fmicb.2021.751746)
Supplement: Supplementary file 1 [file Data_Sheet_1.PDF]

Supplementary Material

Supplementary Table 1. 16S rRNA gene sequence similarities of strains F13^T, F24A^T, F28 and F27^T in comparison to available sequences in the EzBiocloud database from any previous described species.

16S rRNA gene sequence	Hit taxon name	Similarity (%)
Strain F13^T *rrnA* (MH447277)	*Halomicroarcula limicola* YGHS32^T	96.5
Strain F13^T *rrnB* (MH447279)	*Halomicroarcula salina* YGHS18^T	95.7
Strain F24A^T *rrnA* (MH447282)	*Halomicroarcula pellucida* BNERC31^T	95.3
Strain F24A^T *rrnB* (MH447281)	*Haloarcula vallismortis* ATCC 29715^T	96.1
Strain F28 *rrnA* (MH450228)	*Halomicroarcula pellucida* BNERC31^T	95.3
Strain F28 *rrnB* (MH447330)	*Haloarcula vallismortis* ATCC 29715^T	96.2
Strain F27^T *rrnA* (MH447286)	*Halomicroarcula limicola* YGHS32^T	99.2
Strain F27^T *rrnB* (MH447284)	*Haloarcula salaria* HST01-2R^T	94.4

Supplementary Table 2. Differential characteristics between strains F13^T, F24A^T, F28, F27^T and related species of the genus *Halomicroarcula*.

Characteristic	Strain F13^T	Strain F24A^T	Strain F28	Strain F27^T	***Halomicroarcula pellucida* CECT 7537^T**	***Halomicroarcula limicola* JCM 18640^T**	***Halomicroarcula salina* JCM 18369^T**
Morphology	Rods	Rods	Pleomorphic	Rods	Pleomorphic[a]	Pleomorphic[b]	Pleomorphic rods[c]
Colony pigmentation	Red	Pink	Pink	Orange red	Transparent (non-pigmented)[a]	Red[b]	Red[c]
NaCl requirement:							
Range (%)	10-30	15-30	15-30	10-30	20-30[a]	10-30	15-30
Optimum (%)	30	25	25	25-30	25[a]	25	20
Temperature requirement:							
Range (°C)	25-50	25-50	25-50	20-50	25-55[a]	20-50[b]	20-50[c]
Optimum (°C)	37	37	37	37	40[a]	37[b]	37[c]
pH requirement:							
Range	6.0-9.0	6.0-8.5	6.0-8.0	6.0-9.0	6.0-8.5[a]	6.0-8.5[b]	5.5-9.0[c]
Optimum	7.5-8.0	7.5	7.0	7.5	7.0[a]	7.5[b]	7.0[c]
Anaerobic growth with:							
L-Arginine	-	-	-	-	ND	+	+
Potassium nitrate	-	-	-	-	ND	-	-
Dimethyl sulfoxide	-	-	-	-	ND	-	-
Hydrolysis of:							
Gelatin	+	+	+	-	-	-	-
Aesculin	-	+	+	+	+	+	+
Tween 80	-	+	+	-	-	-	-
Production of acid from carbohydrates:							
Arbutin	+	+	+	+	-	-	-

**Characteristic**	**Strain F13ᵀ**	**Strain F24Aᵀ**	**Strain F28**	**Strain F27ᵀ**	***Halomicroarcula pellucida* CECT 7537ᵀ**	***Halomicroarcula limicola* JCM 18640ᵀ**	***Halomicroarcula salina* JCM 18369ᵀ**
L-Citrulline	+	+	+	+	-	+	-
D-Glucose	+	-	+	+	+	+	-
D-Mannitol	-	-	-	-	+	-	-
Utilization as sole carbon and energy source of:							
D-Cellobiose	-	+	+	-	-	+	+
D-Galactose	-	-	-	+	-	+	-
D-Glucose	-	+	+	+	-	+	+
Ribose	-	-	-	+	+	+	+
Sucrose	-	+	+	+	+	ND	ND
Salicin	-	-	-	+	-	-	+
Glycerol	-	-	-	+	-	+	+
D-Sorbitol	-	+	+	+	-	-	+
L-Arginine	+	-	-	-	-	+	+
L-Cysteine	-	-	-	-	+	-	-
L-Methionine	+	-	-	-	-	-	+
Isoleucine	-	-	-	-	+	ND	ND
Valine	-	-	-	-	+	+	+
Citrate	-	+	+	-	-	-	-
Fumarate	-	+	+	+	-	-	+
Propionate	-	-	-	-	+	+	+
Tartrate	-	+	+	-	-	-	-

All data from this study unless otherwise indicated. +, positive; -, negative; ND, not determined. [a]Data from Echigo et al., 2013. [b]Data from Zhang and Cui, 2014. [c]Data from Zhang and Cui, 2015.

Supplementary Table 3. Accession number of the genomes of *Haloarcula* and *Halomicrobium* species used for comparison in this study.

**Reference species**	**Accession number**
Haloarcula amylolytica JCM 13557^T	AOLW01000000
Haloarcula argentinensis DSM 12282^T	AOLX01000000
Haloarcula hispanica ATCC 33960^T	NC_015948
Haloarcula japonica DSM 6131^T	AOLY01000000
Haloarcula marismortui ATCC 43049^T	NC_006396
Haloarcula quadrata DSM 11927^T	RBWW01000000
Haloarcula salaria ZP1-2	RZND01000000
Haloarcula sebkhae JCM 19018^T	BMPD01000000
Haloarcula vallismortis ATCC 29715^T	AOLQ01000000
Halomicrobium katesii DSM 19301^T	AQZY01000000
Halomicrobium mukohataei DSM 12286^T	CP001688
Halomicrobium zhouii CGMCC 1.10457^T	FOZK01000000

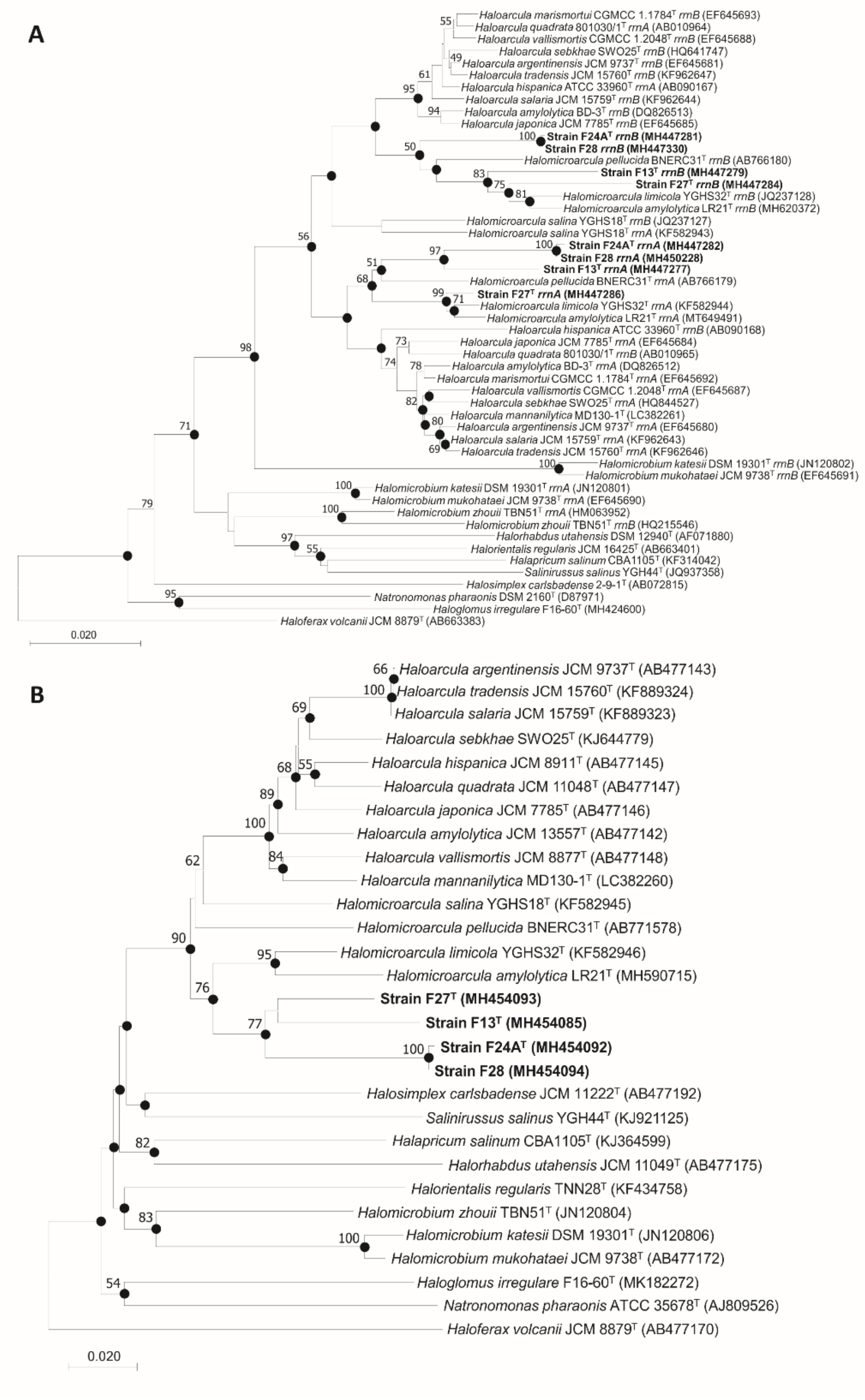

Supplementary Figure 1. Neighbour-joining phylogenetic trees based on A) 16S rRNA gene sequences of strains F13^{T}, F24A^{T}, F27^{T}, F28, members of the genus *Halomicroarcula* and related genera; B) *rpoB´* gene sequences of of strains F13^{T}, F24^{T}, F27^{T}, F28, members of the genus *Halomicroarcula* and related genera.

Filled circles indicate branches that were also supported by the maximum-likelihood algorithm. Sequence accession number are shown in parentheses. Bootstrap values ≥ 50% are shown at branch points. Bar, 0.02 changes per nucleotide position.

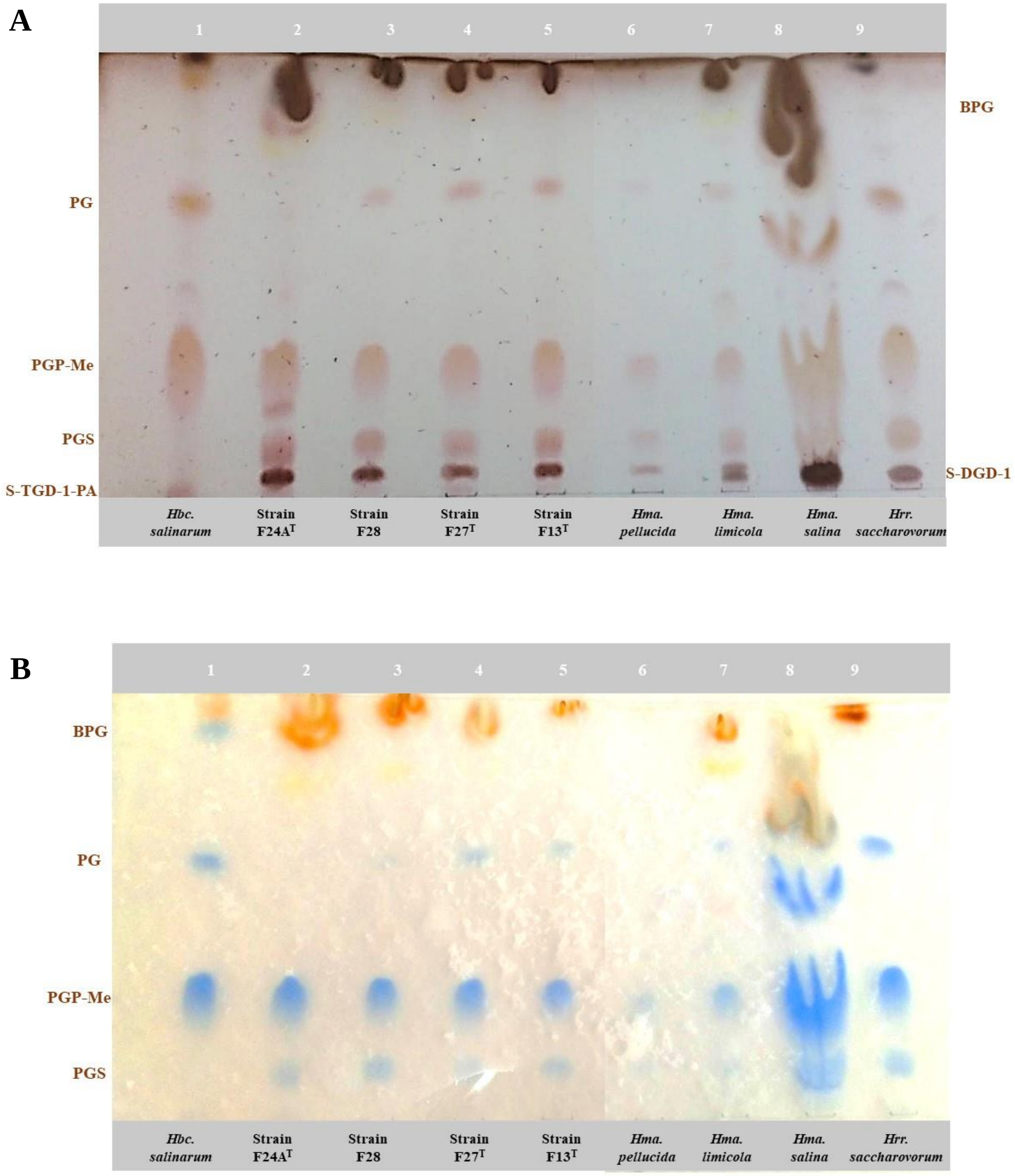

Supplementary Figure 2. High performance thin layer chromatography (HPTLC) of the comparison of the polar lipids (A) and phospholipids (B) profile between *Halomicroarcula* strains and some other haloarchaeal species. The plate was revealed with sulfuric acid 5 % in water, followed charred by heating at 160 °C (A) and with molybdenum blue spray reagent (B).

Abbreviations: BPG, biphosphatidylglycerol; PG, phosphatidylglycerol; PGP-Me, phosphatidylglycerol phosphate methyl ester; PGS, phosphatidylglycerol sulfate; S-DGD-1, sulfated diglycosil diether; S-TGD-1-PA, glycocardiolipin (sulfated triglycosyl diphytanyl archaeol ester linked to phosphatidic acid).

A

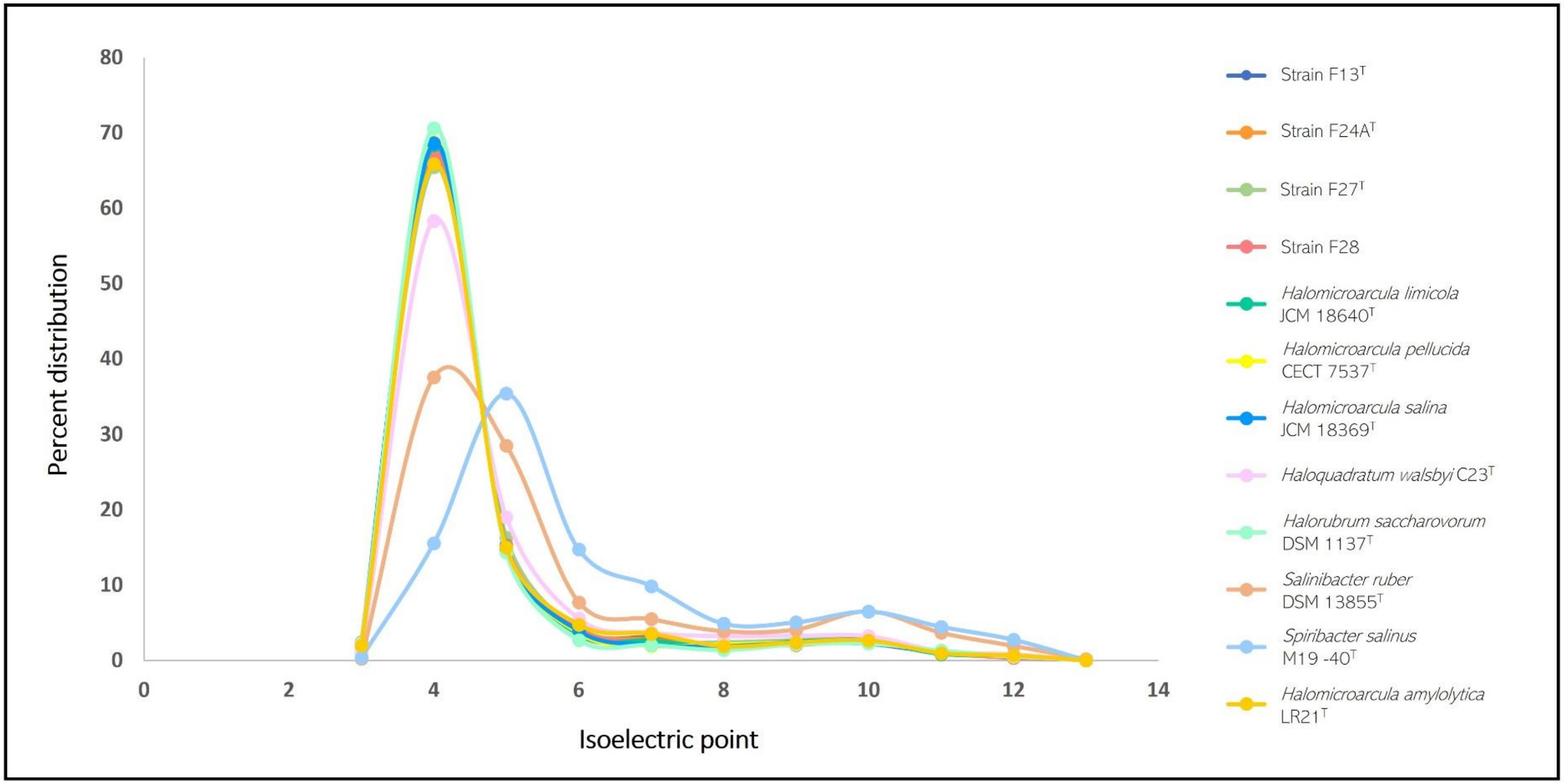

80
70
60
50
40
30
20
10
0
Percent distribution
0
2
4
6
8
10
12
14
Isoelectric point
Strain F13T
Strain F24AT
Strain F27T
Strain F28
Halomicroarcula limicola JCM 18640T
Halomicroarcula pellucida CECT 7537T
Halomicroarcula salina JCM 18369T
Haloquadratum walsbyi C23T
Halorubrum saccharovorum DSM 1137T
Salinibacter ruber DSM 13855T
Spiribacter salinus M19 -40T
Halomicroarcula amylolytica LR21T

B

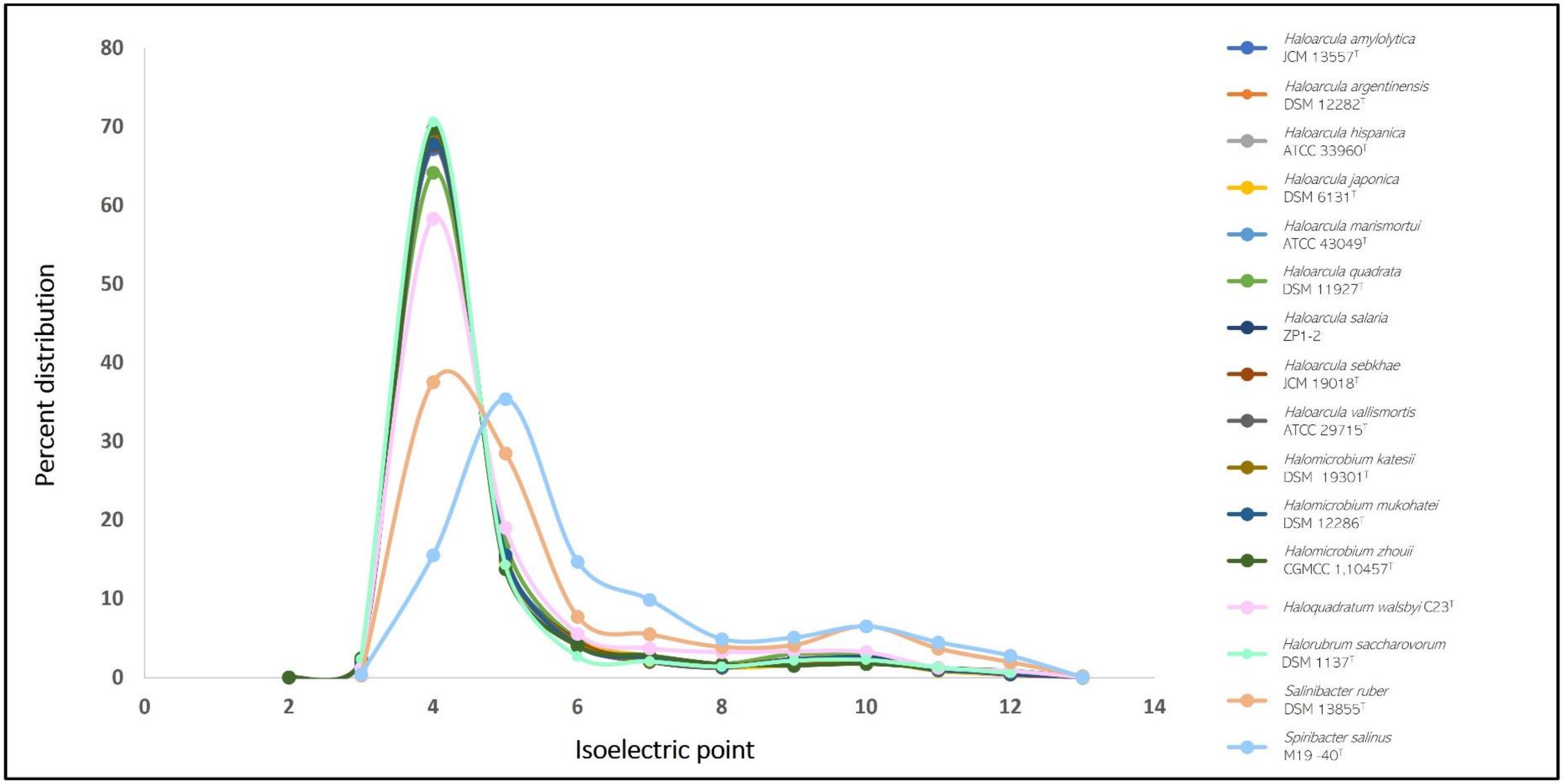

Supplementary Figure 3. Comparison of isoelectric point of predicted proteins for *Halomicroarcula* strains and other prokaryotic species (A) and for *Haloarcula* and *Halomicrobium* species and other prokaryotic species (B), computed for each translated genome and shown as a percentage of distribution.
